# Enhancing Breast Cancer Detection and Classification Using Advanced Multi-Model Features and Ensemble Machine Learning Techniques

**DOI:** 10.3390/life13102093

**Published:** 2023-10-21

**Authors:** Mana Saleh Al Reshan, Samina Amin, Muhammad Ali Zeb, Adel Sulaiman, Hani Alshahrani, Ahmad Taher Azar, Asadullah Shaikh

**Affiliations:** 1 Department of Information Systems, College of Computer Science and Information Systems, Najran University, Najran 61441, Saudi Arabia; msalreshan@nu.edu.sa (M.S.A.R.); shaikhasad@hotmail.com (A.S.); 2 Institute of Computing, Kohat University of Science and Technology, Kohat 26000, Pakistan; kustsameena@gmail.com (S.A.); alizeb@kust.edu.pk (M.A.Z.); 3 Department of Computer Science, College of Computer Science and Information Systems, Najran University, Najran 61441, Saudi Arabia; aaalsulaiman@nu.edu.sa (A.S.); hmalshahrani@nu.edu.sa (H.A.); 4 College of Computer and Information Sciences, Prince Sultan University, Riyadh 11586, Saudi Arabia; 5 Automated Systems and Soft Computing Lab (ASSCL), Prince Sultan University, Riyadh 11586, Saudi Arabia

**Keywords:** breast cancer, Wisconsin Diagnostic Breast Cancer, machine learning, ensemble learning, feature selection, detection, classification

## Abstract

Breast cancer (BC) is the most common cancer among women, making it essential to have an accurate and dependable system for diagnosing benign or malignant tumors. It is essential to detect this cancer early in order to inform subsequent treatments. Currently, fine needle aspiration (FNA) cytology and machine learning (ML) models can be used to detect and diagnose this cancer more accurately. Consequently, an effective and dependable approach needs to be developed to enhance the clinical capacity to diagnose this illness. This study aims to detect and divide BC into two categories using the Wisconsin Diagnostic Breast Cancer (WDBC) benchmark feature set and to select the fewest features to attain the highest accuracy. To this end, this study explores automated BC prediction using multi-model features and ensemble machine learning (EML) techniques. To achieve this, we propose an advanced ensemble technique, which incorporates voting, bagging, stacking, and boosting as combination techniques for the classifier in the proposed EML methods to distinguish benign breast tumors from malignant cancers. In the feature extraction process, we suggest a recursive feature elimination technique to find the most important features of the WDBC that are pertinent to BC detection and classification. Furthermore, we conducted cross-validation experiments, and the comparative results demonstrated that our method can effectively enhance classification performance and attain the highest value in six evaluation metrics, including precision, sensitivity, area under the curve (AUC), specificity, accuracy, and F1-score. Overall, the stacking model achieved the best average accuracy, at 99.89%, and its sensitivity, specificity, F1-score, precision, and AUC/ROC were 1.00%, 0.999%, 1.00%, 1.00%, and 1.00%, respectively, thus generating excellent results. The findings of this study can be used to establish a reliable clinical detection system, enabling experts to make more precise and operative decisions in the future. Additionally, the proposed technology might be used to detect a variety of cancers.

## 1. Introduction

Breast cancer (BC) is a frequent type of cancer in women all over the world, and effective treatment depends on early diagnostic mechanisms. The use of ML and artificial intelligence (AI) to develop precise and effective BC detection and classification methods has gained increasing attention since these technologies were introduced. Among all tumors that affect women, BC has one of the highest incidence and fatality rates. Early BC detection lowers mortality and is essential for extending life expectancy [1]. One of the leading causes of death in women around the world has been recognized as BC. Recent studies have shown that earlier detection is crucial for utilizing effective treatments and lowering the mortality rate among women from BC [2].

Globally, BC accounts for 15% of all malignancies that affect women [3]. It has been reported that BC is the second most prevalent cause of cancer-related death in females, with 1 in 37 individuals dying from the condition [4]. According to the American Cancer Society, BC primarily strikes middle-aged and older women. The median age at the time of BC diagnosis is 62, indicating that half of women diagnosed with BC are 62 years of age or younger. It is noteworthy that only a small percentage of women diagnosed with BC are younger than 45. Furthermore, the World Health Organization (WHO) states that in every country worldwide, BC occurs at any age after adolescence in women but with increasing rates in later life. Preventative screening is essential for the early diagnosis and treatment of BC, and some countries have effectively started screening programs that have reduced the disease burden by almost one third [5].

**Figure 1:**
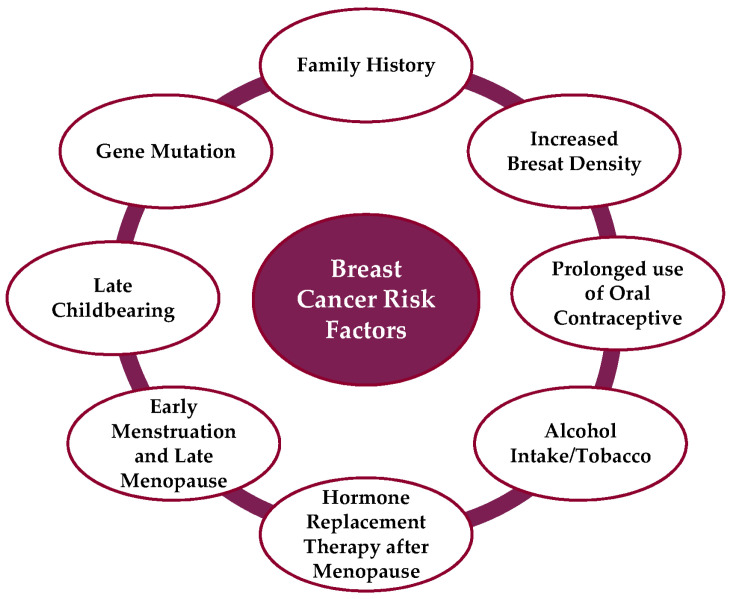
Risk factors for BC disease.

One in eight women will die from BC, making it a common health issue for females [6,7]. With more than 2.3 million cases diagnosed each year, it is the most common cancer among people. According to the WHO, in 95% of the countries worldwide, BC is the main or secondary cause of cancer-related deaths in women. However, there are significant differences in BC survival within and between countries. Nearly 80% of deaths from BC and cervical cancer occur in low- and middle-income countries [8]. However, it is expected that the number of all cancer cases might increase from 14 million to 22 million within the next two decades and then continue to increase gradually each year after that [9]. Because cancer spreads to various sections of the body from its initial site, the mortality rate of all cancers has increased. In 2020, BC claimed the lives of 685,000 people worldwide, affecting 2.3 million women. By the year 2020, BC, which had been detected in 7.8 million women over the preceding five years, was the most prevalent malignancy in the world [9]. In every country, women can develop BC at any age after puberty; however, the incidence rates rise as people age [10]. Figure 1 highlights the cause and risk factors that affect BC disease.

According to the WHO, three out of every ten women diagnosed with BC globally passed away in 2020 [11]. Due to its stealthy development, the majority of BC disorders are detected during routine screening. The incidence, mortality, and survival rates of BC can be influenced by various factors, including environmental conditions, genetics, lifestyle choices, and the composition of the population. When BC is detected and treated promptly, the chances of survival are significantly improved. Early diagnosis and timely intervention play a vital role in improving the prognosis of patients with breast cancer [12]. On 6 March 2023, the WHO published a new global BC Initiative framework [11] that serves as a road map for achieving the goal of preventing 2.5 million deaths from BC by 2040. To accomplish this objective, the new framework, which was published ahead of the World Cancer Day campaign, calls on nations to adhere to the three pillars of health promotion for the early detection, rapid diagnosis, and thorough management of BC.

According to the World Cancer Research Fund International, the ten countries with the greatest incidence of BC in women and the largest number of BC-related fatalities in women in 2020 are outlined in Table 1 and Table 2, respectively [13]. The age-standardized rate (ASR) indicator provides a quick snapshot of the prevalence of disease in a population under a uniform age distribution. Since age significantly affects the likelihood of developing cancer, standardization is necessary when comparing groups of people of different ages. The incidence of BC in women worldwide in 2020 is shown in Table 1. In 2020, Belgium and the Netherlands were the two countries with the highest rates of BC among women. The mortality rates for BC in women worldwide in 2020 are shown in Table 2. In 2020, Fiji was the country with the highest rate of female BC fatalities, followed by Barbados.

AI and ML methods can be trained on various BC data, including medical imaging data such as mammography and ultrasound scans [14,15,16,17]. AI models can accurately forecast and diagnose BC by analyzing images to find patterns and characteristics linked to the disease. Additionally, utilizing various feature selection and extraction methodologies, researchers have recently created several ML models for the diagnosis and classification of BC. The process of choosing the characteristics or variables that are most beneficial for predicting a specific outcome is known as feature selection. Advanced feature selection methods can help a model employ fewer features, increasing its accuracy and efficiency [18]. To tackle the dramatically increasing cancer rate, early detection and ML technologies are widely used for the diagnosis and prognosis of a variety of ailments, including oral cancer, cardiovascular diseases [19,20], lung cancer [21], diabetes [22,23] and BC [24,25,26,27,28]. The promising results achieved have led scientists to explore the possibility of utilizing data mining as a method for predicting BC recurrence.

For BC detection, traditional diagnosis includes biopsy for pathology assessment, and advanced imaging modalities such as mammography, ultrasound, and breast magnetic resonance imaging (MRI) are employed for imaging and initial evaluation. In the context of data mining and ML, breast tumor measurements derived from these imaging techniques are utilized to distinguish between malignant and benign tumors, which is commonly referred to as the BC prediction problem [28,29]. The input features, parameter settings, and model topologies are only a few of the variables that affect classification performance. Finding a successful method to achieve good performance for general classification tasks is still difficult [30]. Since classification outcomes directly impact patient care and safety, BC diagnosis is more crucial than ever. It necessitates strong reliability and robustness in addition to high prediction accuracy, which is another difficulty for data mining experts. Each method has advantages and disadvantages when used for various categorization problems. To capitalize on the strength of individual classifiers, ensemble learning is one of the most widely used techniques. Although many weak bases can be used to build a strong ensemble classifier, research indicates that the base classifiers’ characteristics have an impact on the effectiveness of the ensemble outputs [31,32].

In summary, enhancing BC detection and classification models with advanced EML and feature selection algorithms has the potential to improve the performance of BC diagnosis systems, enabling earlier detection and more effective treatment. In this study, different architectures of ML models, recognized for high classification accuracy, are adapted and combined as an EML model for BC diagnosis in order to compensate for the shortcomings of individual base classifiers and optimize their results. The ML models include logistic regression (LR), the decision tree classifier (DTC), random forest classifier (RFC), support vector classifier (SVC), Gaussian naïve Bayes (GNB), K-neighbors classifier (KNC), the extra trees classifier (ETC), neural network (NN) or multi-layer perceptron (MLP), adaptive boosting classifier (ABC), gradient boosting classifier (GBC), and extreme gradient boosting (XGBC). Additionally, for EML, we used stacking, bagging, voting, and boosting. The three contributions of this study are as follows: (1) the application of sophisticated EML algorithms for the detection and classification of BC; (2) the incorporation of feature selection methods to find the most useful features; and (3) the thorough assessment of our suggested methodology using publicly accessible benchmark WDBC data [33]. WDBC is a modified/processed version of the WBC dataset. It often includes additional features and preprocessing to make it more suitable for ML and diagnostic purposes. On the other hand, the WBC dataset typically refers to the original version of the data, which may contain raw data. Using WDBC, our findings may aid in the development of BC diagnosis tools that are more precise and dependable, ultimately enhancing patient care and treatment results. From the wide pool of accessible variables, feature selection is essential for locating the most pertinent and instructive features. The performance and interpretability of the BC detection and classification model are improved by choosing the most discriminative features, which also helps to decrease the dataset’s dimensionality and lessen the risk of overfitting.

The primary contributions of the suggested technique are as follows:

1. An enhanced model is proposed for the detection and classification of BC into benign and malignant;
2. Advanced EML algorithms are used, including voting, bagging, stacking, boosting, and feature selection techniques;
3. A promising method is developed to enhance the accuracy and reliability of BC detection and classification models using advanced EML algorithms and feature selection approaches;
4. Feature selection technology such as recursive feature elimination (RFE) is used to determine the most informative features;
5. The proposed model has the potential to contribute to the development of BC detection methods that are more precise and dependable, ultimately enhancing patient care and treatment results.

The remainder of this study is structured as follows: In Section 2, related work is discussed; in Section 3, the proposed methodology is designed and described in detail. Section 4 presents the experimental results and in-depth analysis. Finally, Section 5 concludes the proposed study.

## 2. Related Work

Different ML technologies have been developed in the literature to identify BC cases based on clinical data, including tumor sizes, texture behaviors, and homogeneity of cell morphologies [34,35,36,37,38,39]. To detect BC, Naveen et al. [40] designed the EML method, including feature scaling, cross-validation, and various EML models with a bagging technique. They evaluated their proposed method using the prediction accuracy, confusion matrix, and classification report. H. Wang et al. [31] developed an SVM-based EML model for BC detection. Based on the weighted area under the ROC curve, twelve SVMs with different feature optimization rates were built. Wisconsin Breast Cancer (WBC), WDBC, and the Surveillance, Epidemiology, and End Results (SEER) BC datasets from the U.S. National Cancer Institute were used to gauge the effectiveness of the suggested method. The findings demonstrate that, in comparison to five different EML and two common EML techniques, namely ABC and bagging classification tree, the proposed technique has a greater accuracy with a much-reduced variance for the diagnosis of BC. Comparing the proposed model to the best single SVM architecture on the SEER dataset, the latter improves accuracy by 33.34% and lowers variance by 97.89%. Gopal et al. [18] proposed a methodology to perform early BC diagnosis utilizing the Internet of Things (IoT) and ML. The objective of the article was to investigate the application of ML methods in conjunction with IoT devices for predicting BC. The proposed classifier achieved high performance with precision, recall, F-score, and accuracy of 98%, 97%, 96%, and 98%, respectively.

In order to detect BC in its early stages, ref. [41] proposed a heterogeneous EML approach. The suggested method involves stacking to design an EML mechanism utilizing three separate models: KNN, SVM, and DT. It is based on the CRISP-DM process. At K = 20, the EML technique had the lowest log-loss of 0.56 and the highest accuracy of 78%, rejecting the null hypothesis. For the one-tailed *t*-test, which has a lesser significance level at = 0.05, a calculated *p*-value of 0.014 was obtained. Abdar et al. [5] established a two-layer nested EML. The metaclassifiers in their two-layer layered EML use two or three separate classification mechanisms. They used the WDBC dataset for tests, and the K-fold cross-validation mechanism was employed to evaluate the techniques. They analyzed the model’s performance in terms of accuracy, precision, recall, F1-score, ROC, and computational duration of training two-layer nested EML and single models (i.e., Bayes Net and NB). The outcomes showed that the two-layer nested EML is superior to single classifiers, and compared with the majority of earlier efforts, it obtained an accuracy of 98.07%. Uddin et al. [42] divided BC into benign and malignant tumors using a variety of ML classifiers, including RF, NB, SVM, K-NN, DT, ABC, GB, LR, MLP, and voting methods. To gauge the effectiveness of the model, several indices, namely precision, F1-score, accuracy, and sensitivity, were employed. The accuracy of each model was determined to choose the most appropriate one. According to the evaluation, the voting techniques achieved the highest accuracy of approximately 98.77%.

Benbrahim et al. [43] developed an ML-based system using the WBCD dataset and evaluated the classification test accuracy. The findings of their study revealed that the NN had the highest accuracy, with an impressive accuracy rate of 96.49%. Ghiasi et al. [32] applied the RF and extra tree techniques to categorize the type of BC. For the goal of classification, the suggested methods offer a straightforward and effective graphical methodology. To construct a BC diagnosing model, the WBCD delivers actual data that covers the most important criteria. In [44], an ANN model was used to diagnose and predict BC by employing the WBCD and WDBC without using selection algorithms. The ANN exhibited promising performance in categorizing benign and malignant tumors utilizing the WBCD and WDBC datasets.

Khashei et al. [45] established the efficacy of the MLP technique, which was applied to numerous BC samples, and compared its classification rate to the traditional MLP technique. According to the findings, the suggested discrete learning-based MLP model performed better than the MLP across all datasets. The MLP had an average accuracy of 94.70%, which is a 6.95% increase over the accuracy of the conventional MLP technique of 88.54%. Singh et al. [46] used a combination of ML techniques based on AI to develop a prediction model while coupling soft computing approaches. They used WDBC datasets to assess the efficiency of the system, and the results demonstrate that the hybrid algorithm has superior performance in BC classification, with an accuracy of 98.9578%. Using WDBC dataset, Sharma et al. [47] designed an EML with NN and extra trees. Additionally, the suggested approach, which combines a neural network and an extra tree, performs better than existing techniques across a range of performance measures. Dhanya et al. [48] designed an ML-based method using LR, NB, and RF. For feature selection, sequential feature selection, F criteria, RFE, and correlation were taken into consideration. In this analysis, the publicly accessible UCI repository WDBC was used. The findings demonstrate that the RF method provides the best feature selection accuracy.

A major drawback in the current studies is that traditional ML approaches frequently rely on a single model, which can be prone to overfitting when working with complicated and high-dimensional data. Furthermore, complex patterns or relationships in the data may be difficult for conventional approaches to represent. Contrarily, EML methods can overcome these constraints by merging several base models and using the combined intelligence of these models to increase the predicted accuracy and robustness. Enhancing BC detection and classification models with advanced EML and feature selection algorithms has the potential to enhance the accuracy and competence of BC diagnosis systems, allowing for earlier detection and more effective treatment. In this study, various ML models, recognized for high classification accuracy, are adapted and combined as an EML model for BC diagnosis to compensate for the shortcomings of individual base classifiers and optimize their results. The three contributions of this paper include (i) the application of sophisticated EML algorithms for the detection and classification of BC, (ii) the incorporation of feature selection mechanisms to find the most useful features, and (iii) the thorough assessment of our suggested methodology experimented on WDBC dataset [33]. Our findings may aid in the development of BC diagnosis tools that are more precise and dependable, ultimately enhancing patient care and treatment results. Among the wide pool of accessible variables, feature selection is essential for finding the most pertinent and instructive features. The performance and interpretability of the BC detection and classification model can be improved by choosing the most discriminative features, which also helps to decrease the dataset’s dimensionality and lessen the risk of overfitting. In conclusion, a reliable method should be developed to diagnose and monitor BC more accurately.

## 3. The Proposed Study Framework

**Figure 2:**
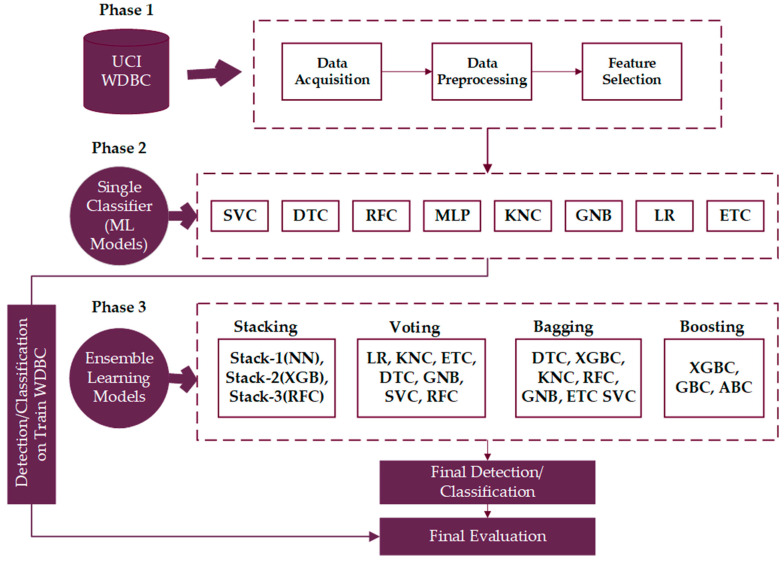
Proposed EML framework.

This section briefly demonstrates the study framework proposed for the detection and classification of BC, as depicted in Figure 2.

### 3.1. Dataset Acquisition and Preprocessing

**Figure 3:**
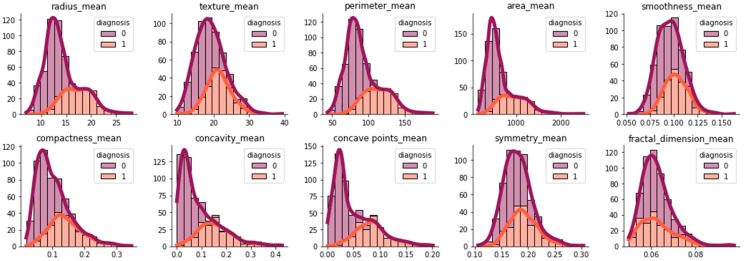
Histogram plots for the distribution of features in a dataset: the normal distribution and positively skewed distribution (0: benign and 1: malignant).

In this study, the Wisconsin Diagnostic Breast Cancer (WDBC) dataset was used for model training. The University of California Irvine (UCI) ML repository provided this dataset [33,49]. It contains 32 tumor characteristics from 569 patients. The features are calculated from a digital image of a breast mass that is aspirated with an FNA. The 32 features include an instance ID, a class label indicating whether each instance has a benign or malignant tumor, and 30 real tumor traits. Ten real-valued parameters were assessed for each cell nucleus in this data sample, and the statistical analysis is presented in Figure 3. As previously stated, the BC diagnosis problem is addressed in this article as a two-type (benign (B) or malignant (M)) classification problem. According to Table 3, the WDBC contains 569 instances, and the target feature holds two types of tumors, namely benign tumors and malignant tumors. There are 357 (62.7%) instances of benign tumors and 212 (37.3%) malignant tumors.

These features are real-valued parameters that are assessed using a digital image of a breast mass aspirate. They depict features of the cell nuclei that are present in the image. From these features, ten instances, namely radius, perimeter, area, smoothness, texture, compactness, concavity, symmetry, concave points, and fractal dimension, were estimated for each cell nucleus [50] and can be seen graphically in Figure 3. Figure 3 depicts the histogram plots for the distribution of features in the dataset by analyzing the normal distribution and positively skewed distribution. For each image, the average of the worst three measurements and their standard errors were calculated, resulting in a repository of 30 real-valued input parameters covering 569 instances. In the dataset, there were no missing values.

### 3.2. Exploratory Data Analysis of the WDBC Dataset

**Figure 4:**
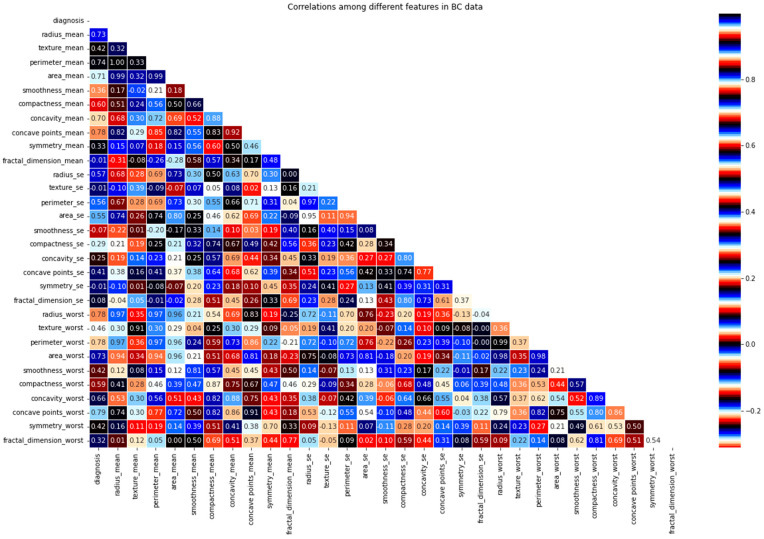
Heatmap of the extracted features applying the Pearson correlation coefficient technique. The dark and light colors in the heatmap scale indicate the negative and positive correlation between each feature, respectively.

**Figure 5:**
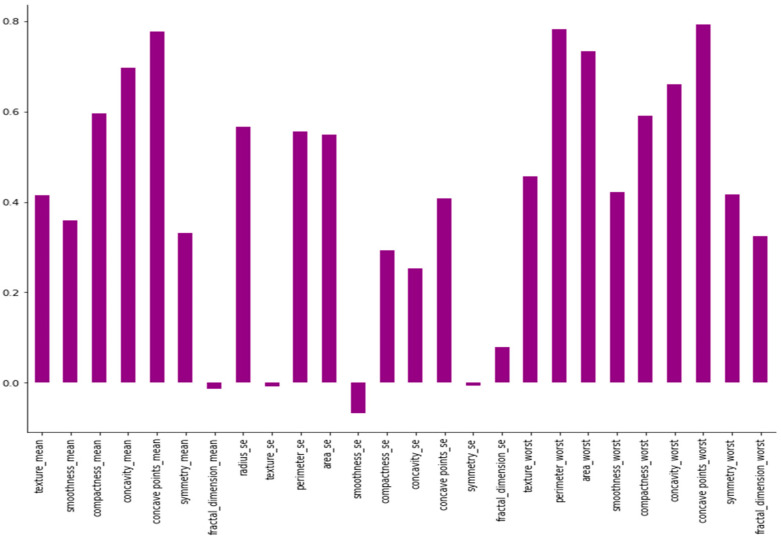
Correlation with the target class.

This section presents an exploratory data analysis by using correlation techniques to properly analyze the data in the WDBC. For this, a heatmap is used, which is valuable when attempting to determine the density of data in a two-dimensional matrix. By conducting a correlation study on two variables, we may determine how closely they are related. Correlation analysis can be used to establish a linear relationship between two variables. Figure 4 shows a two-dimensional matrix where each value in the dataset is represented by a distinctive color.. Values in two-dimensional cells greater than 0 indicate a positive correlation between attributes, while values less than 0 indicate a negative correlation between attributes. In the heatmap, a correlation that is near 0 implies a weak or no relationship between the variables, while a correlation that is positive or negative reflects a strong dependency or strong inverse dependency. Figure 5 shows the correlation of features with the target class.

One method to quantify this link is to use the Pearson correlation coefficient, which assesses the linear implication between two variables. It ranges in value from −1 to 1 as follows: A value of 1 signifies a positive linear correlation between two different dataset features, a value of 0 indicates there is no linear correlation between two separate dataset features, and a value of −1 directs a negative linear correlation.

### 3.3. Feature Selection Using Recursive Feature Elimination

**Figure 6:**
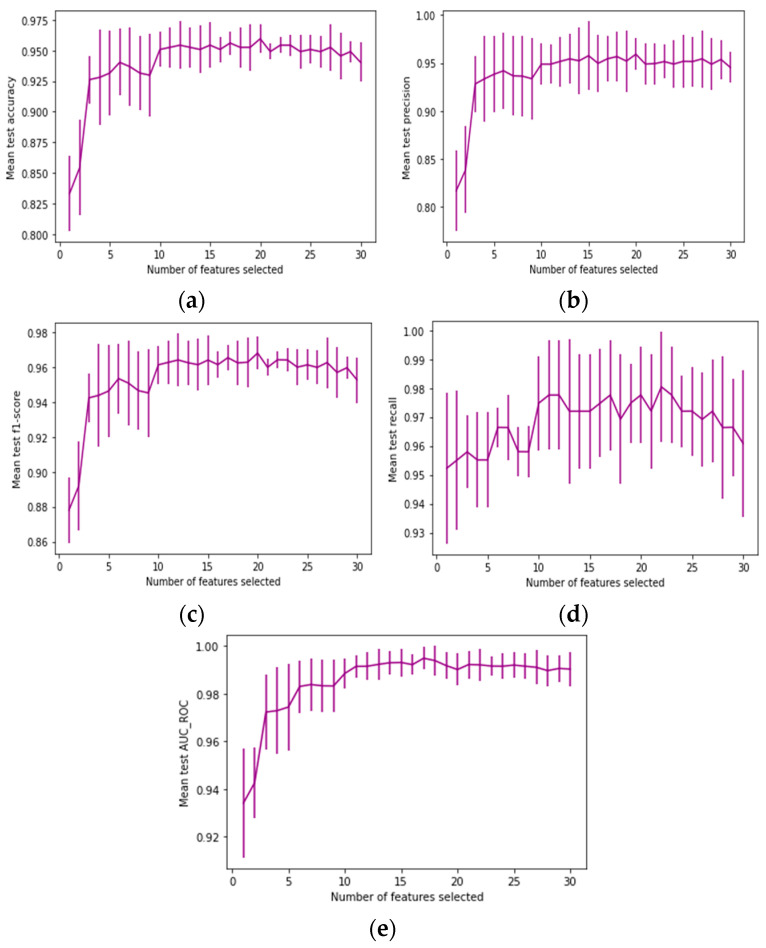
RFE with cross-validation for different features: (**a**) accuracy; (**b**) precision; (**c**) recall; (**d**) F1-score; and (**e**) AUC/ROC.

Although there are numerous potential causes of BC, it is very difficult to determine the precise environmental factors and other causes involved. Nevertheless, these factors are important in predicting the development of cancer. We can accomplish our objective of estimating the risk of occurrence of BC using EML and common diagnosis data. Numerous patient characteristics are present in BC data; however, not all of these characteristics help predict cancer. Feature selection techniques are helpful in these situations to select the pertinent feature set. In this work, the RFE technique was used to obtain the most significant prediction features. This procedure is popular because it is simple to apply and efficient in choosing pertinent features from training datasets for predicting target variables and screening out irrelevant features. Using the RFECV, the chosen features were visualized, and the number of features in the WDBC data was determined, along with the cross-validated scores, as shown in Figure 6. The cross-validated scores were calculated after the RFE object was established. The percentage of correctly categorized samples was optimized using the accuracy, precision, recall, F1-score, and AUC/ROC scoring mechanisms. In this scheme, a method with 22 features, which is the genuine generative model, was found to be the most effective. A plateau of identical scores (the same mean value and overlapping error bars) for 4 to 22 chosen features can also be seen in Figure 6. This occurs when associated features are introduced. Indeed, depending on the cross-validation method, the RFE’s optimal model choice may fall within this range. Keeping non-informative features causes overfitting, which is risky for the statistical efficiency of the models because the test accuracy decreases over 22 selected features.

### 3.4. Ensemble Machine Learning Models

Ensemble learning refers to an ML approach in which multiple models, often referred to as “weak learners”, are trained to address the same problem and then combined to achieve enhanced results. The fundamental principle behind this methodology is that by appropriately combining these weaker models, we can obtain more precise and resilient models. The subsection below describes the EML models utilized in this study.

#### 3.4.1. Bagging

Bagging is used when a decision tree’s variance needs to be reduced. The idea is to extract a few smaller datasets from the training sample, which are selected at random with replacement. Then, each data subset is employed to create its decision tree, and as a result, we have an ensemble of different models. Using the average of all the assumptions from several decision trees is more effective than using only one.

Bagging involves training multiple instances of the same model (often referred to as weak learners) using different subsets of the training data, which are randomly sampled with replacement (bootstrap samples). The final prediction is achieved by averaging (for regression) or voting (for classification) the individual predictions of each method. Bagging helps reduce overfitting and improves model stability by reducing variance.

#### 3.4.2. Voting

Voting, also known as majority voting, is a simple EML method that combines the predictions of multiple independent models (can be different algorithms or variations) for classification tasks. Among the individual model predictions, the final prediction is determined by a majority vote. It is particularly useful when the base models have different strengths and weaknesses, as it can take advantage of their diversity to improve overall performance.

#### 3.4.3. Boosting

Boosting is an iterative EML technique through which multiple weak learners are sequentially designed, with each new learner focusing on the mistakes of its predecessors. In boosting, the base classifiers are trained sequentially, and each classifier allocates more weight to the occurrences misclassified by preceding classifiers. This allows for emphasizing challenging data points and gradually improving the overall performance of the ensemble.

#### 3.4.4. Stacking

Stacking is an EML model that involves combining multiple base algorithms by training a higher-level “meta-model” on their predictions. The base algorithms make predictions on the input data, and their outputs are used as features to train the meta-model. Stacking aims to leverage the strengths of different models and learn to consider their predictions optimally, potentially leading to better generalization and performance.

In the proposed work, we used ML-based models, namely LR, SVC, KNC, XGBC, GNB, DTC, and RFC, as the base layers (models). For the meta-layer (mode), we used NN, RFC, and XGBC to make predictions on the output generated by base layers as features.

### 3.5. Performance Metrics

To determine the superiority of each model, a comparison of their performance indices is necessary. These indices include accuracy, sensitivity, specificity, precision, F1-score, and ROC/AUC score [51]. Each metric offers valuable insights into various aspects of the model’s performance. The performance metrics employed in this study can be calculated using the following equations (Equations (1)–(5)):

(1) $$Precision=\frac{TP}{\left(FP+TP\right)}$$

(2) $$Accuracy=\frac{TP+TN}{\left(TP+TN+FN+FP\right)}$$

(3) $$Sensitivity=\frac{TP}{\left(FN+TP\right)}$$

(4) $$Specificity=\frac{TN}{\left(FN+TN\right)}\times 100\%$$

(5) $$F1\text{-}Score=2\times \frac{\left(Recall\times Precision\right)}{\left(Recall+Precision\right)}$$

In Equations (1)–(5), the true positive (TP) is the number of BC cases among the instances detected accurately as benign and malignant tumors. While the true negative (TN) is the number of cases among the instances that are detected as neither benign nor malignant BC. False positive (FP) is the number of instances that were detected as benign BC but were assigned in the WDBC as malignant BC, and false negative (FN) represents the number of instances that were incorrectly detected as benign when they were actually malignant breast tumors. Accuracy measures how many instances of BC tumors are correctly detected, as defined in Equation (2). Precision is calculated as the ratio of true-positive cases (WDBC data detected as malignant BC tumors) to the total number of cases detected as malignant BC tumors by the model, as mentioned in Equation (1). According to Equation (3), sensitivity is the ratio of the WDBC data correctly detected as malignant BC tumors using the model to all actual malignant BC tumors, while the F1-score is a harmonic mean of precision and sensitivity (Equation (5)). It provides a balanced measure that combines both precision and sensitivity, offering a comprehensive assessment of the model’s performance (Equation (5)).

**Figure 7:**
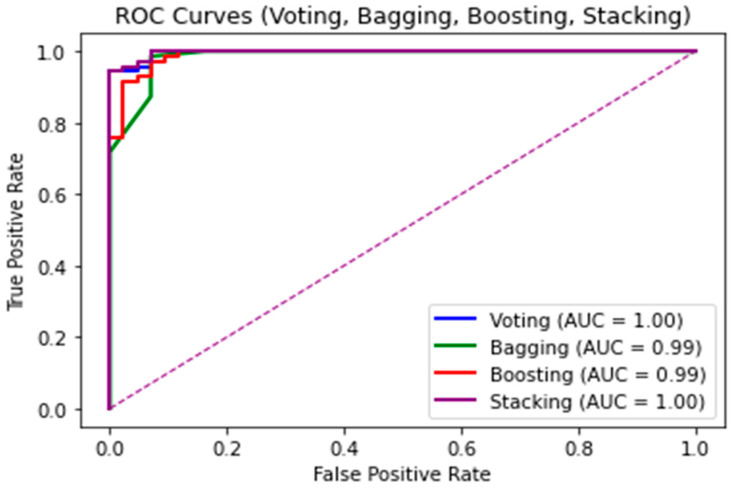
ROC curve of the best models.

The AUC-ROC curve, shown in Figure 7, provides a visual representation of the proposed EML model performance. It assesses the sensitivity and specificity (Equations (3) and (4)) of the binary classifier through ROC analysis. ROC signifies a probability curve, and AUC quantifies the extent of separability [51], indicating the model’s ability to distinguish between classes.

Additionally, this study explores the K-fold cross-validation techniques. Cross-validation helps to estimate the model’s performance on unseen data. In K-fold, the dataset is split into K subsets. For this study, the commonly used value of 10 for K was applied to evaluate the models’ performance. The analysis revealed the most promising outcome after cross-validation, based on performance metrics, enabling the selection of the best model among the various classifiers (see Table 4 and Table 5).

## 4. Results and Discussion

In this section, we compare the predictive abilities of the EML, including stacking, voting, bagging, and boosting models. The experiments were conducted using Python (3.8 version) as the programming language, and Anaconda (Anaconda Inc., Austin, TX, USA) as the software tool, along with built-in packages, namely Sklearn, Numpy, Pandas, Matplotlib, Keras, and Seaborn, to perform the experiments and evaluate the results. Using the WDBC dataset, several tests were carried out, and the results were meticulously analyzed to explore the actual improvements in order to further assess the proposed model for adaptation. For this, we used a multi-model feature ensemble learning technique incorporating EML, RFE methods, and statistical approaches on the WDBC dataset to detect and classify BC into benign and malignant tumors. The accuracy was assessed by applying a 10-fold cross-validation method. The final accuracy was then calculated by averaging the accuracy obtained in each of the 10 iterations. Using cross-validation ensures a more robust assessment of the model’s performance and reduces the risk of overfitting or bias in the evaluation process. It allows for a better estimation of how the method will perform on unseen data, making the accuracy metric more reliable and meaningful.

The proposedtechniques were also compared with other ML and EML algorithms in terms of accuracy, precision, specificity, sensitivity, F1-score, and AUC/ROC on WDBC by applying a 10-fold cross-validation mechanism. Table 4 displays the experimental results derived for the WBCD dataset. In terms of performance, Table 4 shows that the stacking algorithm outperformed the voting, bagging, and boosting methods. Among the stacking methods with the best accuracy, the stack-1(NN) model had the highest accuracy (99.89%). Furthermore, Figure 7 shows the ROC curve of the best EML model, namely voting (1.00%), stacking (1.00%), bagging (99.00%), and boosting (99.00%), when compared to the EML developed by Abdar et al. [5] and the SVC by Naji et al. [52]. In the ROC metric, evaluated using the AUC, a perfect score of 1.00% was achieved for voting and stacking, respectively. Furthermore, the results show that both the stacking and voting EML models achieved a perfect TPR (1.00%), meaning that they correctly classified all benign and malignant cases. On the other hand, the bagging and boosting models achieved a TPR of 99.00%, demonstrating their high accuracy in detecting and classifying benign and malignant cases. This illustrates that the proposed method has an excellent discriminative ability to distinguish between benign and malignant cases, regardless of the detection threshold. A perfect AUC score indicates that these algorithms can accurately diagnose the cases of BC (benign and malignant cases) with no FPR or FNR predictions. Previous research studies [5,52] have shown promise in tackling the BC diagnostic challenge by combining additional EML techniques and using additional parameters and various data samples. Thus, these challenges should be investigated for further improvement.

The stacking ensemble model had better accuracy than any individual traditional model by effectively integrating numerous base classifiers. The procedure proposed in this study is a novel approach for improving the performance of BC diagnosis and early identification. According to Table 4, the best average accuracy was found to be 99.89%, and the sensitivity, specificity, F1-score, precision, and AUC/ROC of the stack-1(NN) model were 1.00%, 0.999%, 1.00%, 1.00%, and 1.00%, respectively.

**Figure 8:**
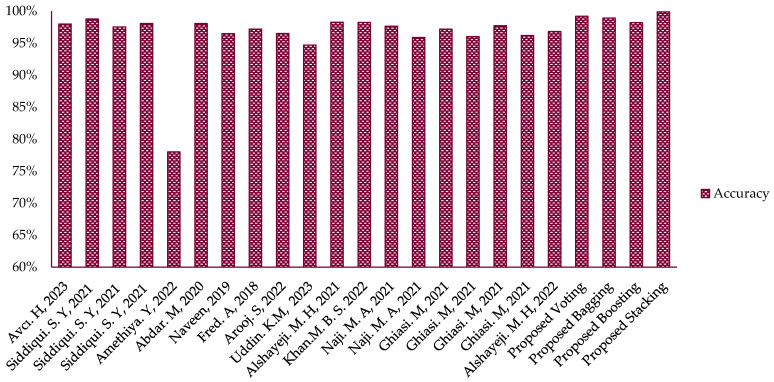
Comparison of accuracy among the existing work and proposed models [6,18,29,33,39,40,41,43,45,45,51,52,53].

Additionally, the highest precision, sensitivity, F1-score, and ROC/AUC achieved using the best model (stack-1(NN)) were 1.00%, 1.00%, 1.00%, and 1.00%, respectively. The accuracy and specificity were 99.89% and 99.90%, respectively. The accuracy rates achieved using the proposed models, namely voting, bagging, and boosting, were 99.20%, 98.93%, and 98.20%, respectively (Table 4). The outcomes of this study demonstrate that the proposed stack-1 model outperforms many state-of-the-art classifiers. Table 5 presents a comparison between the performance results of the proposed model and the most recent EML models that concentrate on utilizing the WDBC dataset for BC diagnosis. With the help of metrics like classification accuracy, specificity, sensitivity, recall, precision, and F1-score, the effectiveness of the suggested model was assessed. The proposed approach provides better outcomes when compared with current existing studies, as evidenced by the simulation results presented in Table 5 and Figure 8.

The purpose of this comparison is to demonstrate the superiority of the proposed model over the existing EML models such as Bayes Net and NB [5], RF + SVC [30], KNN + SVM + DT [41], LR + SVM [42], and NN + ETC [47], as well as ML techniques such as MLP [18], KNN, MLP, SVM and ANN [34], NN [43], MLP [45], SVC [52], XGB [53], and SVM and RF [54] in terms of detection and classification performance on the WDBC dataset. Hence, these state-of-the-art techniques have shown promising results in addressing the challenges of detecting and classifying BC at an early stage; however, the working mechanisms of each approach are different and therefore should be investigated for further improvement. The existing techniques were compared with the proposed EML, which includes stacking, bagging, boosting, and voting. This comparison aims to identify challenges for potential improvement. In terms of the effectiveness of the obtained results of the proposed models, a comprehensive comparison between the proposed method and the other existing methods available in the literature is presented in Table 5. Figure 8 depicts a comparison of accuracy among the existing work and proposed models. Notably, the proposed stack-1(NN) exhibited superior performance when compared to various well-known techniques, including voting, bagging, boosting, and many other existing models.

Experimental simulations, empirical findings, and statistical analysis show that the suggested method is more effective and advantageous for BC detection and categorization. Considering the relevant literature, the proposed method was also compared with existing EML approaches [5,30,41,42,47] as well as ML techniques [18,34,43,45,52,53,54] in terms of accuracy. This comparison is graphically presented in Figure 8, where the suggested model’s accuracy is contrasted with the accuracy of the state-of-the-art approaches. The findings support the assertion that the new method holds promise for enhancing the accuracy and efficacy of BC detection and classification.

In conclusion, the stack-1(NN) model stands out as a promising EML method that has state-of-the-art performance in a wide range of detection and classification challenges, including BC diagnosis, with an impressive accuracy of 99.89%. Its outstanding performance underscores its potential as a valuable EML model in the field of medical diagnostics and highlights the promising impact of ML algorithms on healthcare outcomes and decision systems.

### 4.1. Implications

In this study, we focused on improving the effectiveness and performance accuracy of BC detection and classification by integrating advanced multi-model features and EML approaches. Our study aims to reduce false positives and false negatives, improve feature extraction and selection, and use the strength of various algorithms for more accurate diagnosis and detection of BC disease. An efficient model can lead to enhance BC detection and classification, medical decision making, and better patient outcomes in BC care. However, in medical decision making, accurate and reliable detection and classification are of utmost importance, as they directly impact patient outcomes and treatment plans. By incorporating the proposed strategy, medical professionals can benefit from improved diagnostic accuracy, leading to better patient care and management.

Overall, the implications of this research could help to advance the field of BC diagnostics, potentially leading to more efficient, accurate, and prompt medical interventions.

### 4.2. Limitations

The effectiveness of this work may be influenced by the quality and diversity of the training samples, potential challenges in feature integration, and the interpretation of the EML techniques. Furthermore, the suggested EML model’s application in real-world healthcare environments, as well as its versatility across diverse patient demographics and imaging modalities, may bring additional problems and constraints to address.

### 4.3. Future Work

For future research, the implementation of different AI technologies based on deep learning and ensemble techniques needs to be explored. In this case, deep neural networks, long short-term memory, BERT, and convolutional neural networks with different optimization techniques and ensemble methods can be considered. Nevertheless, these approaches provide even more challenges when it comes to interpreting the findings that should support the choices/decisions made by healthcare professionals.

## 5. Conclusions

BC is a frequent type of cancer among women all around the world, and effective treatment depends on early diagnostic mechanisms. The classification and diagnosis of BC present significant problems to the medical profession. For women, BC is a leading cause of death. Cancer patients’ greatest concern is recurrence, which might lower their quality of life. Because BC is one of the main causes of death in women, early identification is essential. One of the greatest challenges in the field of healthcare research is the timely and accurate detection of various diseases. Importantly, in this work, a new EML technique was developed to enhance BC early diagnostic classification algorithms. The performance of EML strategies, which combine multiple independent learning algorithms, has frequently been found to be either better than or on par with that of a single base classifier. As a result, it has become more well known and has proved effective in the field of ML. One of the most significant issues that needs to be resolved is the use of EML strategies. This is why, in this study, we used different K-folds to assess how well EML approaches performed. The stacking ensemble model had better accuracy than any individual traditional model by effectively integrating numerous base classifiers. The procedure proposed in this study offers a novel method for improving the performance of BC detection and early identification. Overall, the best average accuracy was determined as 99.89%, and the sensitivity, specificity, F1-score, precision, and AUC/ROC of the stack-1(NN) model were 1.00%, 0.999%, 1.00%, 1.00%, and 1.00%, respectively. The comparison results demonstrate that the stack-1 model outperforms the alternative approaches in terms of precision, sensitivity, accuracy, F1-score, AUC, and specificity performance metrics.

## Figures and Tables

**Table 1:** BC incidence rates in women worldwide: source [13].

Rank	Country	Number	ASR/100,000
	World	2,261,419	47.8
1	Belgium	11,734	113.2
2	The Netherlands	15,725	100.9
3	Luxembourg	497	99.8
4	France	58,083	99.1
5	France, New Caledonia	185	99.0
6	Denmark	5083	98.4
7	Australia	19,617	96.0
8	New Zealand	3660	93.0
9	Finland	5228	92.4
10	US	253,465	90.3

**Table 2:** BC death rates in women worldwide: source [13].

Rank	Country	Number	ASR/100,000
	World	684,996	13.6
1	Barbados	111	42.2
2	Fiji	184	41.0
3	Jamaica	637	34.1
4	Bahamas	80	31.0
5	Papua New Guinea	847	27.7
6	Somalia	1189	27.2
7	Mali	1425	26.6
8	Dominican Republic	1577	26.4
9	Syria	1946	26.2
10	Samoa	21	25.6

**Table 3:** Total number of WDBC instances divided into benign and malignant tumors.

BC Types	Total Number of Instances	Ratio (%)
Benign tumors	357	62.7%
Malignant tumors	212	37.3%
Total	569	100.0%

**Table 4:** Mean score and standard deviation of accuracy, precision, sensitivity, specificity, F1-score, and ROC/AUC for the proposed models.

Ensemble Model	Model	Accuracy	Precision	Sensitivity	Specificity	F1-Score	ROC/AUC
Stacking (Base Layer)	GNB	0.973 ± 0.157	0.978 ± 0.018	0.964 ± 0.022	0.946 ± 0.018	0.952 ± 0.02	0.979 ± 0.017
	SVC	0.977 ± 0.023	0.972 ± 0.014	0.975 ± 0.020	0.969 ± 0.021	0.961 ± 0.026	0.965 ± 0.025
	KNC	0.988 ± 0.105	0.973 ± 0.011	0.985 ± 0.020	0.981 ± 0.020	0.977 ± 0.030	0.996 ± 0.010
	DTC	0.916 ± 0.017	0.935 ± 0.031	0.937 ± 0.028	0.930 ± 0.029	0.946 ± 0.040	0.950 ± 0.021
	RFC	0.969 ± 0.014	0.969 ± 0.010	0.979 ± 0.017	0.977 ± 0.018	0.978 ± 0.015	0.989 ± 0.010
	ETC	0.951 ± 0.011	0.963 ± 0.012	0.966 ± 0.025	0.965 ± 0.250	0.968 ± 0.025	0.983 ± 0.011
	LR	0.960 ± 0.016	0.974 ± 0.025	0.954 ± 0.021	0.964 ± 0.023	0.951 ± 0.011	0.973 ± 0.015
Stacking (Meta-Layer)	Stack-1(NN)	0.9989 ± 0.010	1.00 ± 0.001	1.00 ± 0.012	0.999 ± 0.010	1.00 ± 0.001	1.00 ± 0.0001
	Stack-2 (XGB)	0.986 ± 0.009	0.998 ± 0.008	0.998 ± 0.007	0.999 ± 0.006	1.00 ± 0.002	1.00 ± 0.0001
	Stack-3 (RFC)	0.990 ± 0.006	0.993 ± 0.003	0.993 ± 0.005	0.990 ± 0.008	1.00 ± 0.0013	1.00 ± 0.0001
Voting	Voting (LR, KNC, ETC, DTC, GNB, SVC, RFC)	0.992 ± 0.010	0.988 ± 0.008	0.990 ± 0.006	0.989 ± 0.009	0.997 ± 0.011	1.00 ± 0.003
Boosting	XGBC	0.982 ± 0.009	0.983 ± 0.010	0.980 ± 0.007	0.987 ± 0.011	0.986 ± 0.009	0.991 ± 0.010
	GBC	0.977 ±0.011	0.973 ± 0.019	0.968 ± 0.022	0.983 ± 0.008	0.973 ± 0.010	0.983 ± 0.005
	ABC	0.959 ± 0.024	0.969 ± 0.013	0.969 ± 0.019	0.951 ± 0.011	0.969 ± 0.014	0.969 ± 0.020
Bagging	DTC	0.946 ± 0.040	0.943 ± 0.014	0.933 ± 0.024	0.932 ± 0.014	0.951 ± 0.011	0.964 ± 0.022
	XGBC	0.949 ± 0.033	0.943 ± 0.015	0.943 ± 0.014	0.946 ± 0.040	0.949 ± 0.033	0.954 ± 0.030
	KNC	0.965 ± 0.025	0.960 ± 0.016	0.969 ± 0.020	0.977 ± 0.019	0.973 ± 0.020	0.973 ± 0.010
	RFC	0.989 ± 0.017	0.985 ± 0.008	0.977 ± 0.023	0.986 ± 0.008	0.988 ± 0.010	0.993 ± 0.005
	GNB	0.937 ± 0.028	0.937 ± 0.028	0.942 ± 0.024	0.930 ± 0.029	0.933 ± 0.028	0.952 ± 0.012
	ETC	0.968 ± 0.025	0.972 ± 0.015	0.983 ± 0.011	0.985 ± 0.009	0.980 ± 0.007	0.993 ± 0.004
	SVC	0.978 ± 0.019	0.978 ± 0.018	0.974 ± 0.020	0.981 ± 0.007	0.974 ± 0.020	0.990 ± 0.008

**Table 5:** Detection and classification accuracy comparison with existing work conducted using the WDBC dataset.

	Work	Model	Accuracy	Precision	Sensitivity	Specificity	F1-Score	AUC/ROC
Existing state-of-the-art methods	[18]	MLP	98.00%	0.98	0.97	-	0.96	-
	[42]	LR + SVM	98.77%	0.9883	0.9854	-	0.9868	-
	[42]	MLP	97.54%	0.9755	0.9718	-	0.9736	-
	[42]	SVM	98.07%	0.9828	0.9761	-	0.9792	-
	[41]	EML	78.00%	-	-	-	-	-
	[5]	EML	98.07%	0.9810	0.9810	-	0.9810	0.9760
	[43]	NN	96.49%	-	-	-	-	-
	[52]	SVC	97.20%	0.98	0.94	-	0.96	0.966
	[30]	RF + SVM	96.50%	-	-	-	-	-
	[45]	MLP	94.70%	-	-	-	-	-
	[51]	XGB	98.73%	99.48	99.43	-	-	0.989
	[53]	XGB	98.24%	-	-	-	-	-
	[54]	SVM	97.66%	0.98	0.95	-	0.97	-
	[54]	RF	95.90%	0.97	0.91	-	0.94	-
	[34]	KNN	97.19%	-	-	-	-	-
	[34]	MLP	96.03%	-	-	-	-	-
	[34]	SVM	97.72%	-	-	-	-	-
	[34]	ANN	96.19%	-	-	-	-	-
	[47]	ABC	96.82%	0.960	0.9548	0.9743	0.9558	-
Proposed work	Voting	LR, KNC, ETC, DTC, GNB, SVC, RFC, XGB	99.20%	0.988	0.990	0.989	0.997	1.00
	Bagging	RFC	98.93%	0.985	0.977	0.986	0.988	0.993
	Boosting	XGBC	98.20%	0.983	0.980	0.987	0.986	0.991
	Stacking	Stack-1(NN)	99.89%	1.00	1.00	0.999	1.00	1.00

## Data Availability

The datasets used to support the experimental outcomes of this study are available from the direct link in the dataset citations.
